# Evaluation of a *PlexZyme*-Based PCR Assay and Assessment of COVID-19 Surge Testing Throughput Compared to Cobas SARS-CoV-2

**DOI:** 10.3390/pathogens10091088

**Published:** 2021-08-26

**Authors:** Todd M. Pryce, Erin J. Haygarth, Jessica Bordessa, Peter A. Boan

**Affiliations:** 1 PathWest Laboratory Medicine WA, Department of Clinical Microbiology, Fiona Stanley Hospital, Murdoch, WA 6150, Australia; erin.haygarth@health.wa.gov.au (E.J.H.); jessica.bordessa@health.wa.gov.au (J.B.); peter.boan@health.wa.gov.au (P.A.B.); 2 Department of Infectious Diseases, Fiona Stanley Fremantle Hospital Group, Murdoch, WA 6150, Australia

**Keywords:** SARS-CoV-2, PlexZyme, high-throughput

## Abstract

Reliable high-throughput methods are required for the detection of severe acute respiratory coronavirus 2 (SARS-CoV-2). We evaluated the new research use only (RUO) SpeeDx *PlexZyme* SARS-CoV-2 components (Plex) compared to the Roche cobas SARS-CoV-2 assay (cobas). A collection of positive (*n* = 214) and negative samples (*n* = 201) was tested in parallel comparing Plex with cobas. The overall agreement comparing the qualitative outcomes was 96.9%. Using an in-house quantitative PCR method, correlation comparing Plex ORF1ab to cobas ORF1a was r^2^ = 0.95. The median Plex ORF1ab change in target copy number compared to cobas ORF1a was +0.48 log_10_ copies/mL respectively. Inter- and intra-assay reproducibility of each assay was compared, including a limit-of-detection study. Reproducibility was comparable; however cobas was more sensitive than Plex by 1-log dilution. Throughput was evaluated during a COVID-19 testing surge of 4324 samples in a 30-h period. Plex demonstrated less hands-on time per reportable result (19% decrease) and increased throughput (155% increase of 102 results/hour) compared to cobas (40 results/hour). Our study demonstrates good qualitative and quantitative correlation of Plex compared to cobas and that Plex is well-suited for high throughput testing.

## 1. Introduction

As of 1 July 2021, more than 180 million cases of Coronavirus disease 2019 (COVID-19) have been declared worldwide, resulting in 3.9 million deaths [1]. Diagnostic tools are essential to manage the current COVID-19 pandemic and reliable, high-throughput laboratory tests are required [2]. These tools are the strategic cornerstone to mitigate SARS-CoV-2 spread, facilitating the early diagnosis, isolation of infected individuals and clearance of essential personnel to continue to work [3]. Since the 19th of March 2020, our laboratory has performed more than 300,000 tests with the majority of testing performed using the cobas SARS-CoV-2 assay (cobas) (Roche, Basel, Switzerland) [4]. Other essential diagnostic services such as blood-borne virus testing (BBV) on the cobas 6800 instrument (Roche) were maintained despite the additional SARS-CoV-2 workload. As with other laboratories, we implemented SARS-CoV-2 testing in addition to other diagnostic services, placing tremendous strain on laboratory resources. To alleviate the SARS-CoV-2 workload and provide additional routine testing capacity on the cobas 6800 instrument, we sought to utilise other instruments in our laboratory for SARS-CoV-2 testing. These include two MagNAPure 96 instruments (Roche) for nucleic acid extraction combined with two LightCycler 480 thermal cyclers (Roche). We also sought to implement a dual-target test to mitigate the risk of single-nucleotide polymorphisms, which has been reported for the cobas SARS-CoV-2 pan-*Sarbecovirus* E-gene target [5]. A 384-well thermal cycling method was considered advantageous to maximise testing throughput; hence, a 384-well liquid handler was also a mandatory requirement. To combine these elements, we evaluated the RUO *PlexZyme* CoV-2 (RdRp/ORF1ab) components (Plex) (SpeeDx, Sydney, Australia). Plex targets the conserved open reading sequences (ORF1ab) and the RNA-dependant RNA polymerase gene (RdRp). The *PlexZyme* technology utilises a PlexPCR approach and touch-down PCR for superior specificity and multiplexing capability [6]. The method utilizes the PlexPrep liquid handler (SpeeDx) to prepare and dispense master mix to a 384-well PCR plate then transfer nucleic acids from up to four 96-well MagNAPure 96 output plates. Amplification and detection in the 384-well format takes 82 min irrespective of the number of samples. Since implementation of cobas testing, stored SARS-CoV-2-positive samples (naso-oropharyngeal samples in virus transport medium or universal transport medium) were used to evaluate the *PlexZyme*-based assay and associated RdRp and ORF1ab gene targets to detect SARS-CoV-2. Particular attention to throughput capability of Plex compared to cobas was assessed and significant throughput advantages were noted. As a result of this evaluation the Plex assay was implemented into routine use. On the 31 January 2021, the Western Australian Government announced a lockdown which resulted in a surge of COVID-19 testing. From 31 January 2021 1826 h to 2 February 2021 0050 h (30 h), our laboratory tested and reported 4324 tests combining cobas and Plex methods. We outline the testing performed during this period detailing the cobas and Plex testing strategy for high-throughput COVID-19 testing. We present the first manufacturer-independent evaluation of the *PlexZyme*-based method compared to cobas and examine the throughput capabilities of both methods during a surge of COVID-19 testing.

## 2. Materials and Methods

### 2.1. Routine Sample Testing

All samples were previously tested using the cobas SARS-CoV-2 assay (Ref. 09175431190) as the primary screening method. All samples were naso-oropharyngeal swabs collected in either Copan UTM-RT media (Brescia, Italy), CITOSWAB (Citotest Scientific Jiangsu, Haimen, China) or Virus Transport Media (VTM) [7]. Thermal pre-treatment of the sample was performed before cobas testing [4]. Briefly, 600 µL of the sample transport media was transferred to a cobas omni secondary tube (Ref. 06438776001) and thermally treated for 75 °C for 15 min in a QBD4 dry block heater (Grant Instruments, Cambridge, UK). Samples were tested on the cobas 6800 instrument without delay. According to the manufacturer’s instructions, a sample is reported as SARS-CoV-2 detected if ORF1a is positive with or without a positive E-gene. In the case of positivity with E-gene alone, the result should be reported as SARS-CoV-2 presumptive positive. Our laboratory confirms all single-target positive cobas results with an alternative method. Briefly, samples positive for ORF1a and E-gene were defined as SARS-CoV-2 detected. Samples positive for a single cobas target (either ORF1a or E-gene) were reflexively tested using Xpert Xpress SARS-CoV-2 (Xpert) (Cepheid, Sunnyvale, CA, USA) from the original sample (not thermally treated). Samples positive for at least one different target compared to cobas were defined as SARS-CoV-2 detected (cobas ORF1a positive with Xpert N2 positive and/or Xpert E-gene positive, or cobas E-gene positive and Xpert N2 positive). All other results including Xpert not detected results were considered equivocal for SARS-CoV-2 and repeat collections were performed. All SARS-CoV-2 detected samples were stored at −80 °C as aliquots from the remaining original sample. All negative samples were stored at 4 °C in the original transport media tube.

### 2.2. Validation Panel Characterisation and Preparation

A validation panel consisting of SARS-CoV-2 detected samples (*n* = 214; positive sample group) and SARS-CoV-2 not detected samples (*n* = 201; negative sample group) was prepared from the stored samples above. No equivocal SARS-CoV-2 samples were used in the validation panel as these were not considered true-positives for the purposes of method comparison. To prepare positive samples for this study a 0.2 mL aliquot from each sample was diluted with 1.8 mL of a naso-oropharyngeal matrix (1:10 dilution). The matrix consisted of pooled cobas negative patient samples (oro-nasopharyngeal swabs) in VTM. The pooled matrix tested negative with cobas and Xpert. All dilutions were prepared in cobas omni secondary tubes and stored at −80 °C until testing. Negative samples were stored at 4 °C and were not diluted.

### 2.3. Cobas and Plex Parallel Testing

All samples in the validation panel were tested with cobas and *PlexZyme* CoV-2 (RdRp/ORF1ab) components (Ref. 7130010) in a blinded fashion. Five parallel runs were performed. Samples were transferred to MagNAPure 96 deep-well plates for nucleic acid extraction (Plex method: see below) then the remaining sample loaded on the cobas 6800 without delay. Cobas testing was performed following the instructions for use (no thermal treatment). Thermal treatment prior to cobas testing was not compared to Plex as thermal treatment has shown to reduce detectable viral RNA [4,8,9] and not validated by the manufacturer. All samples tested with Plex were extracted using MagNA Pure 96 DNA and Viral NA small volume kit (Roche) using the Pathogens Universal 200 (version 4.0) protocol. The AccuPlex SARS-CoV-2 Reference Material (AccuPlex; Ref. 0505-0126) (SeraCare Life Sciences, Milford, MA, USA) was used as a positive control and VTM was used as a negative control. A sample input volume of 200 µL and an elution volume of 50 µL with on-board Plex internal control addition (20 µL per sample) was used. The internal control consisted of 36 µL of Plex IC RNA in 3564 µL of phosphate buffered saline. The master mix consisted of 5 µL Plex Master Mix (2×), 0.1 µL RTase (100×), 0.2 µL RNase Inhibitor (50×), 0.5 µL CoV-2 Mix and 1.7 µL Nuclease-free water for a total of 7.5 µL per reaction. The PlexPrep liquid handler (SpeeDx) was utilised for distribution of master mix (7.5 µL) and addition of nucleic acid extracts (2.5 µL) to the LightCycler 480 384-well reaction plate (Roche). Amplification and detection were performed using the LightCycler 480 II instrument (Roche). Thermal cycling conditions were 48 °C for 10 min (reverse transcriptase); 95 °C for 2 min (enzyme activation); 10 cycles of a touchdown sequence consisting of 95 °C for 5 sec, 61 °C for 30 sec followed by 0.5 °C reduction per cycle for 30 sec to 56 °C (touchdown); 40 cycles of 95 °C for 5 sec, 52 °C for 50 sec (quantification cycling) with a fluorescence acquisition at 510 nm (ORF1ab), 580 nm (RdRp), 610 nm (internal control); and 40 °C for 30 sec (cooling). Data were analysed on the LightCycler 480 using Abs Quant/Second derivative max method to obtain the cycle of quantification (*C_q_*) for Plex. The Plex internal control was used to assess sample extraction validity (<26.0 *C_q_*). The AccuPlex positive control (<25 *C_q_*) and VTM negative control (negative) was used to assess run validity. The results for cobas were interpreted as detected, presumptively detected and negative as in Section 2.1. Plex results were detected if either target was positive and negative if both targets were negative.

### 2.4. Quantitative Standards and Analysis

Quantitative standards were prepared from a commercially available SARS-CoV-2 standard (Exact Diagnostics, Fort Worth, TX, USA). Exact Diagnostics SARS-CoV-2 standard (ExactD×) contains ORF1ab, E-gene and RdRp synthetic RNA transcripts quantitated to 200,000 copies/mL using Bio-Rad Digital Droplet PCR (Bio-Rad, Hercules, CA, USA). We pooled multiple ExactDx vials and 10-fold serially diluted in molecular grade water (G-Biosciences, St. Louis, MO, USA) to prepare six standards over the range of 0.30 to 5.30 log_10_ copies/mL. Each standard was tested in triplicate with cobas (no thermal treatment) and Plex. The mean *C_t_* value at each concentration was used to calculate ORF1a and E-gene standard curves and regression for cobas and similarly for ORF1ab and RdRp for Plex. At least two replicates at each dilution were required to be positive to be included in the standard curve and regression analysis. The regression formulas were used to calculate the respective target copy number for each assay and for all positive samples and controls for the entire study.

### 2.5. Assessment of Intra- and Inter-Assay Reproducibility

As part of the QConnect programme an external positive control (EQC) was also performed routinely to monitor inter-assay reproducibility (Optitrol NAT SARS-CoV-2; DiaMex, Heidelberg, Germany) [10]. A single lot number of EQC (DM20119) was tested over 20 consecutive runs for cobas and Plex. Intra-assay reproducibility was tested with 10 replicates of the EQC in a single cobas and Plex run. The qualitative outcomes and *C_t_*/*C_q_* values were recorded. The quantitative results were calculated from the quantitative standards as described above.

### 2.6. Assessment of the Lower Limit of Detection

A high-titre SARS-CoV-2 positive patient sample (alpha variant) was diluted with the naso-oropharyngeal matrix diluent used for the comparative evaluation. The sample was calibrated approximately to 6.00 log_10_ copies/mL using the cobas ORF1a target from the ExactDx standard curve. Ten-fold serial dilutions were prepared (10 replicates of each) with the naso-oropharyngeal matrix diluent covering the range of 0.00 to 5.00 log_10_ copies/mL (1 to 1.00 × 10^5^ copies/mL). Five replicates of each standard were tested in parallel with cobas and Plex. The qualitative outcomes and *C_t_*/*C_q_* values were recorded. The quantitative results were calculated from the quantitative standards as described above. Assessment of the lower limit of detection was performed by log dilution comparison.

### 2.7. Assessment of Hands-on Time and Throughput

Total hands-on time was assessed for each assay from the sample receipt into the laboratory to reporting the final result. This included the time to register samples in the laboratory information system. Assessment of throughput was performed by retrospective analysis of data captured from the laboratory information system during a period of surge testing. The time period was 1824 min (30.4 h) of testing from 31 January 2021 1826 h to 2 February 2021 0050 h.

### 2.8. Data Analysis

A contingency table was prepared to assess overall agreement between cobas and Plex with 95% confidence intervals (95% CIs) using Westgard QC 2 × 2 contingency calculator (Westgard QC, Madison, WI, USA). Linear regression was performed comparing cobas ORF1a and Plex ORF1ab using log_10_ copies/mL. Quantitative results for cobas ORF1a and Plex ORF1ab (log_10_ copies/mL) were compared and the median and standard deviation were calculated for comparison. The mean and standard deviation was calculated for the EQC. All statistical analyses were performed by Excel (Microsoft, Redmond, WA, USA) and MedCalc v15.4 (New York City, NY, USA).

## 3. Results

All raw results (*C_t_*/*C_q_* values) including the calculated quantitative results for this study are presented in the Supplementary Material.

### 3.1. Comparative Evaluation

The qualitative results of the validation panel comparing cobas to Plex are summarised in Table 1. The overall agreement for a Plex detected result (ORF1ab positive ± RdRp positive or vice versa) or not detected result (ORF1ab and RdRp negative), compared to a cobas detected (ORF1a positive ± E-gene positive), not detected (ORF1a and E-gene negative) or presumptive result (ORF1a negative and E-gene positive) was 96.9% (402/415; CI 94.7%–98.2%). Five samples (156, 173, 174, 194 and 196) were detected with Plex that were cobas presumptive. From the Supplementary Material these samples showed cobas E-gene *C_t_* values ranging from 35.60 to 38.06 corresponding to 2.81 to 1.79 log_10_ copies/mL. All of these samples were Plex single-target positive results. Four of these samples were Plex RdRp positive and one sample was Plex ORF1ab positive. The Plex RdRp *C_q_* values for these samples ranged from 24.62 to 28.17 corresponding to 2.94 to 1.85 log_10_ copies/mL. The Plex ORF1ab *C_q_* value for sample 174 was 25.00 corresponding to 2.93 log_10_ copies/mL. Plex detected SARS-CoV-2 in sample 201 with an RdRp *C_q_* of 27.17 (ORF1ab negative) corresponding to 2.16 log_10_ copies/mL (cobas was negative for this sample). Cobas detected 12 samples as presumptive (E-gene only) that were Plex negative. From the Supplementary Material these samples showed cobas E-gene *C_t_* values ranging from 35.09 to 40.70 corresponding to 3.02 to 0.70 log_10_ copies/mL. All of the *C_t_* values observed for discordant results were considered late PCR amplification and detection of SARS-CoV-2. Following the manufacturer’s instructions for reporting results for the positive sample group, 85.0% (182/214) were SARS-CoV-2 detected with cobas (presumptive results excluded) compared to 87.8% (188/214) with Plex. Overall, cobas reported 3.2% more dual-target results than Plex (83.6%; 179/214 for cobas compared to 80.4%; 172/214 for Ple×). All cobas samples with an ORF1a *C_t_* < 31.37 (3.63 log_10_ copies/mL) and E-gene *C_t_* < 33.58 (3.64 log_10_ copies/mL) were Plex ORF1a and RdRp positive (dual-target). No unexpected Plex target discordant samples were observed (negative despite low cobas *C_t_* value).

### 3.2. Quantitative Standards and Assessment of the Lower Limit of Detection

The *C_t_*/*C_q_* values for each target concentration and standard curves for cobas and Plex are shown in the Supplementary Material. Cobas ORF1a demonstrated R-squared (r^2^) value of 0.98 over the range of 1.30 to 5.30 log_10_ copies/mL (5 standards). E-gene demonstrated r^2^ value of 0.98 over the range of 1.30 to 5.30 log_10_ copies/mL (5 standards). Plex ORF1ab demonstrated an r^2^ value of 1.0 over the range of 3.30 to 5.30 log_10_ copies/mL (3 standards). RdRp demonstrated r^2^ value of 0.99 over the range of 2.30 to 5.30 log_10_ copies/mL (4 standards). Cobas limit of detection of the ExactDx ORF1ab and E-gene targets was quantitatively similar. Plex limit of detection of the ExactDx ORF1ab and RdRp targets were different, with RdRp detecting an additional dilution (2.30 log_10_ copies/mL). For the lower limit of detection study using the clinical sample, cobas E-gene was more sensitive than ORF1a with detection of 4/5 replicates at 1.00 log_10_ copies/mL compared to 1/5 replicates for ORF1a. Plex RdRp was also more sensitive than ORF1ab with detection of 3/5 replicates at 2.00 log_10_ copies/mL compared to 1/5 replicates for ORF1ab. Cobas was more sensitive than Plex by at least 1-log dilution overall.

### 3.3. Cobas ORF1a and Plex ORF1ab Correlation

The correlation between log_10_ copies/mL values obtained with Plex ORF1ab compared to cobas ORF1a for all ORF positive samples (*n* = 172) was r^2^ = 0.95. The median quantitative change for Plex ORF1ab compared to cobas ORF1a was +0.48 log_10_ copies/mL. The correlation plots and all raw data are shown in the Supplementary Material.

### 3.4. Assessment of Intra- and Inter-Assay Reproducibility

The *C_t_*/*C_q_* values for each target concentration and standard curves for cobas and Plex are shown in the Supplementary Material, including the results comparing the EQC intra- and inter-assay reproducibility of Plex to cobas. Intra-assay and inter-assay variation for cobas was no more than ±0.38 log_10_ copies/mL and ±0.25 log_10_ copies/mL, respectively. Intra-assay and inter-assay variation for Plex was no more than ±0.36 log_10_ copies/mL and ±0.34 log_10_ copies/mL, respectively. Overall, the reproducibility of the EQC showed less than ±0.5 log_10_ copies/mL variation for both assays. Comparing the mean quantitative results for all EQC results (intra- and inter-assay), we observed a mean difference of 0.01 log_10_ copies/mL between Plex ORF1ab (mean 4.47 log_10_ copies/mL) and Plex RdRp (4.48 log_10_ copies/mL). The mean difference for cobas was greater at a mean difference of 0.59 log_10_ copies/mL between cobas ORF1a (mean 4.27 log_10_ copies/mL) and cobas- E-gene (4.86 log_10_ copies/mL).

### 3.5. Assessment of Hands-on Time and Throughput

Table 2 shows a summary of the surge testing period with cobas and Plex over a 30-h period. Thirteen cobas runs were performed for a total result output of 1222 samples resulting in 40 reportable results per hour. Hands-on-time was 2.1 samples per minute. In contrast, thirty-three MagNAPure 96 extractions were performed, with 15 PlexPrep runs of 188 samples per run and 3 PlexPrep runs of 94. The total result output of Plex was 3102 samples resulting in 102 reportable results per hour. Hands-on time was 1.7 samples per minute. The combined result output was 4324 samples resulting in 142 reportable results per hour with a hands-on time of 3.8 samples per minute. We did not detect any SARS-CoV-2 positive samples during the testing period.

## 4. Discussion

We report the first manufacturer-independent evaluation of the RUO SpeeDx *PlexZyme* SARS-CoV-2 components. Our study aimed to thoroughly evaluate the Plex compared to cobas. We also evaluated the throughput and hands-on time of each assay during a routine COVID-19 testing surge. For the comparative evaluation a direct parallel comparison of Plex with cobas using undiluted original patient material would have been ideal. However, following initial routine testing and our SARS-CoV-2 surveillance testing, the residual sample volume was limited to conduct parallel re-testing with sufficient sample remaining for future research. To overcome this, we diluted all positive samples for this investigation with a pooled oro-nasopharyngeal matrix. The closest representation to an original sample was maintained with this approach. However, the potential disadvantage of this method is that some original samples may be at the lower limit of detection prior to dilution. As such, samples testing negative with both methods are likely as the concentration of SARS-CoV-2 in sample population tested has shifted 1-log closer to (or beyond) the lower limit of detection. The greatest advantage of this approach is sufficient volume to test both methods in parallel, rather than compare the test method results to the retrospective results of a comparative method. Qualitative and quantitative result discrepancies caused by RNA degradation due to storage and freeze-thawing are minimised. A more direct comparison of test performance is possible, with experimental outcomes more likely to be directly attributable to analytical differences. The other advantage is an initial comparative assessment can be made in terms of assay sensitivity of SARS-CoV-2 detection. With our chosen experimental approach, the overall agreement for all samples tested with a positive result for any target in this study was 96.9%. Cobas E-gene was more sensitive than cobas ORF1a (consistent with the manufacturer’s claims) and potentially more sensitive than Plex on initial assessment. However, Plex has the added advantage of two SARS-CoV-2 specific targets, whereas cobas has one specific target and a pan-*Sarbecovirus* target. In this case, the overall correlation in our study between both assays for a SARS-CoV-2 detected result (according to the manufacturer’s instructions for reporting results, excluding cobas presumptive detected results) was 99%. Regardless of which target is positive, we confirm all single-target results using Xpert in routine practice. In this study, we observed 20 single-target results (3 ORF1a and 17 E-gene) for cobas (10.0%; 20/199) and 16 single target results (1 ORF1ab and 15 RdRp) for Plex (8.5%; 16/188). Hence, the percentage of single target results was similar. Xpert has two alternative gene targets compared to Plex; therefore, the Plex/Xpert workflow has a greater likelihood of a definitive SARS-CoV-2 detected result, than the cobas/Xpert workflow with E-gene present in both assays. One other advantage of testing as many representative samples as possible is we were able to assess Plex test performance (target detection specificity) across as many infective SARS-CoV-2 strains as possible, albeit not all genotyped. The failure of a PCR target due to mutations within the primer and/or probe binding regions would severely impair efforts to prevent and control community transmission of SARS-CoV-2 [11]. Issues with target detection have been reported in the literature with cobas E-gene [5] and Xpert N2 region [11,12,13]. We did not observe any unexpected target failures or significantly late *C_q_* values for Plex in the positive sample group when analysing the target regression analysis. Overall, we conclude that Plex performs comparably to cobas for the qualitative detection of SARS-CoV-2.

Our initial observation that cobas was more sensitive than Plex was subsequently confirmed with the lower limit study and testing with the ExactDx standards. However, a key point of difference between patient samples and the standards is the lack of an oro-nasopharyngeal matrix in the standards. We considered diluting the standards in the same oro-nasopharyngeal matrix to control for matrix effects, but we were concerned about nucleases and degradation of the standards which have been calibrated with digital-droplet PCR by the manufacturer. We diluted in nuclease-free water instead and noted a 2-log reduction in the sensitivity of ExactDx ORF1ab detection for Plex ORF1ab compared to cobas ORF1a. However, we observed only a 1-log reduction in sensitivity with Plex ORF1a when multiple replicates of a patient sample were used to assess the lower limit of each assay. We speculate that the nucleic acid matrix of the clinical naso-oropharyngeal specimen may act as an RNA carrier to enhance its recovery during the MagNAPure 96 extraction process. Other reasons for the differences in analytical sensitivity include initial sample volume (400 µL aspirated for cobas compared to 200 µL for Plex), total PCR reaction volume (52 µL for cobas compared to 10 µL for Plex) and template volume (27 µL for cobas, compared to 2.5 µL Plex). We conclude that the Plex approach is a sensitive assay given half the sample volume is extracted and one-tenth of the template used for PCR.

Cycle-threshold values are often used for correlation but should not be used for direct comparisons between assays of different types due to variation in the sensitivity (limit of detection), chemistry of reagents, gene targets, cycle parameters, analytical interpretive methods, sample preparation and extraction techniques [14]. We performed a comparison between cobas and Plex using a quantitative approach. The purpose was to investigate the extent of commutability for the detection of ORF and compare the test performance of target detection, whilst making comparisons with cobas. We found Plex had a smaller difference for the quantitation ORF1ab compared to RdRp than cobas (ORF1a compared to E-gene) for all positive patient samples assessed (n = 172). The smaller difference in Plex target quantitation was also observed for the reproducibility study of the EQC. Overall, Plex demonstrated more consistent results for target quantitation than cobas. We also found a difference in the quantitation of ORF using the ExactDX ORF1ab as a reference (median change +0.48 log_10_ copies/mL). This difference in commutability should not be inferred as a difference in sensitivity, but rather differences for the quantitation of the ExactDx ORF1ab target. The possible reasons for the difference are broad, but may include assay-specific characteristics such as extractions efficiency of standard material compared to patient samples, primer/probe binding efficiency, variable cycling conditions, PCR product size and fragmentation of the target. We included this type of analysis to determine if there were major quantitative differences between the assays for ORF detection. The median difference of <0.5 log is not unusual, especially when compared to other well-established quantitative BBV diagnostic assays which are calibrated to International Standards [15]. A quantitative method using the First WHO International Standard for SARS-CoV-2 RNA [16] is currently being developed in our laboratory for cobas and Plex. This approach will be more useful for lower limit of detection comparisons and clinical studies, especially with prospective parallel testing of clinical samples.

Based on the correlation with cobas and excellent analytical test performance we implemented Plex with the understanding that the Plex workflow has higher throughput capabilities than cobas. This was in-part due to the lack of availability of the cobas 6800 instrument allocated to BBV testing, but more importantly due to availability of our MagNAPure 96 instruments and the installation of the PlexPrep 384-well liquid handler. Multiple 96-well nucleic acid output plates (up to 4) can be used to build a 384-well plate (368 samples) for result turnover in less than 3 h (extraction-to-result) for the first plate, then again every 1.5 h. The throughput capabilities were verified during a single surge event lasting 30 h (4324 reportable results) resulting in a 155% increase in result output with Plex compared to cobas. More reportable results per hour was achievable with a 19% decrease in hands-on-time per reportable result due to the larger volume of tests performed, particularly the 188 samples per run workflow. The sample-to-result cobas workflow is a major advantage though with the ability to load three runs (282 samples) and walk-away. However, we did not verify the throughput capabilities of both methods at full utilisation (continuous operation) as the laboratory had a 5-h down-time period for staff and instrument maintenance operations. Finally, we encourage laboratories to perform their own internal cost analysis particularly for surge testing when operating costs are at their peak. Our laboratory identified considerable savings to consumable costs implementing Plex (cost data not shown).

Detection of SARS-CoV-2 with the cobas 6800 assay has demonstrated to be a sensitive and reliable sample-to-result method [17,18]. In conclusion, the results of the first manufacturer-independent evaluation of Plex on a well-characterised panel of 214 positive and 201 negative samples, has shown that *PlexZyme*-based approach is a reliable assay for the qualitative detection of SARS-CoV-2 in oro-nasopharyngeal samples when compared to cobas. Discordant results were related to single target positives at low concentrations. Our study showed that Plex has high-throughput capabilities and when combined with cobas, represents a solid laboratory testing approach to the increasing testing demands brought to Microbiology by the COVID-19 pandemic.

## Figures and Tables

**Table 1:** Results of the comparative evaluation of the cobas compared to Plex ^a^.

	No of Samples with the Following Result by Cobas			
Plex Result	Detected (Presumptive) ^b^	Not Detected	Total	SARS-CoV-2 Overall Agreement (%) (95% CI) ^d^
Detected ^c^	182 (5)	1	188	96.9 (94.7–98.2)
Not detected	0 (12)	215	227	
Total	199	216	415	

^a^ Results of the comparative evaluation for all samples with interpretation of discordant results given in the Results and Discussion. ^b^ All cobas ORF1a positive results and/or E-gene positive (presumptive). Presumptive results in parentheses. ^c^ Any Plex ORF1ab positive/RdRp positive result. ^d^ Indicates overall agreement for Plex detected/not detected results compared to cobas detected/not detected results (including presumptive results).

**Table 2:** COVID-19 surge testing comparing cobas to Plex over a 30-h period.

Assay Workflow	Number of Runs	Number of Samples per Run	Total Number of Samples Tested	Total Result Output	Results per Hour ^a^	Hands-on Time (Samples per Min) ^b^
cobas	13	94	1222	1222	40	2.1
Plex	15	188	2820	3102	102	1.7
Plex	3	94	282			
Combined total				4324	142	3.8

^a^ Based on total time duration commencing 31 January 2021 1826 h to 2 February 2021 0050 h (1824 min). ^b^ Based on the total result output.

## Supplementary Materials

The following are available online at https://www.mdpi.com/article/10.3390/pathogens10091088/s1, Table S1: all raw results (Ct/Cq values) including the calculated quantitative results for this study are presented in the Supplementary Material.

## References

[1] Ecdc.europa.eu. COVID-19 Situation Update Worldwide, as of Week 52 2020. https://www.ecdc.europa.eu/en/geographical-distribution-2019-ncov-cases.

[2] Caruana G., Croxatto A., Coste A.T., Opota O., Lamoth F., Jaton K., Greub G. (2020). Diagnostic strategies for SARS-CoV-2 infection and interpretation of microbiological results. Clin. Microbiol. Infect..

[3] Centers for Disease Control and Prevention. Implementation of Mitigation Strategies for Communities with Local COVID-19 Transmission. https://www.cdc.gov/coronavirus/2019-ncov/community/community-mitigation.html.

[4] Pryce T.M., Boan P.A., Kay I.D., Flexman J.P. (2021). Thermal treatment of nasopharyngeal samples before cobas SARS-CoV-2 testing. Clin. Microbiol. Infect..

[5] Artesi M., Bontems S., Göbbels P., Franckh M., Maes P., Boreux R., Meex C., Melin P., Hayette M.P., Bours V. (2020). A Recurrent Mutation at Position 26340 of SARS-CoV-2 Is Associated with Failure of the E Gene Quantitative Reverse Transcription-PCR Utilized in a Commercial Dual-Target Diagnostic Assay. J. Clin. Microbiol..

[6] Mokany E., Tan Y.L., Bone S.M., Fuery C.J., Todd A.V. (2013). MNAzyme qPCR with Superior Multiplexing Capacity. Clin. Chem..

[7] Centers for Disease Control and Prevention, Atlanta GA Preparation of Viral Transport Medium (SOP#: DSR-052–03). https://www.cdc.gov/coronavirus/2019-ncov/downloads/Viral-Transport-Medium.pdf.

[8] Burton J., Love H., Richards K., Burton C., Summers S., Pitman J., Easterbrook L., Davies K., Spencer P., Killip M. (2021). The effect of heat-treatment on SARS-CoV-2 viability and detection. J. Virol. Methods..

[9] Chen H., Wu R., Xing Y., Du Q., Xue Z., Xi Y., Yang Y., Deng Y., Han Y., Li K. (2020). Influence of different inactivation methods on severe acute respiratory syndrome coronavirus 2 RNA Copy Number. J. Clin. Microbiol..

[10] QConnect. https://www.nrlquality.org.au/qconnect.

[11] Hasan M.R., Sundararaju S., Manickam C., Mirza F., Al-Hail H., Lorenz S., Tang P. (2021). A Novel Point Mutation in the N Gene of SARS-CoV-2 May Affect the Detection of the Virus by Reverse Transcription-Quantitative PCR. J. Clin. Microbiol..

[12] Ziegler K., Steininger P., Ziegler R., Steinmann J., Korn K., Ensser A. (2020). SARS-CoV-2 samples may escape detection because of a single point mutation in the N gene. Euro. Surveill..

[13] Wang R., Hozumi Y., Yin C., Wei G.W. (2020). Mutations on COVID-19 diagnostic targets. Genomics.

[14] Bustin S., Mueller R., Shipley G., Nolan T. (2021). COVID-19 and Diagnostic Testing for SARS-CoV-2 by RT-qPCR—Facts and Fallacies. Int. J. Mol. Sci..

[15] Preiksaitis J.K., Hayden R.T., Tong Y., Pang X.L., Fryer J.F., Heath A.B., Cook L., Petrich A.K., Yu B., Caliendo A.M. (2016). Are we there yet? Impact of the first international standard for cytomegalovirus DNA on the harmonization of results reported on plasma samples. Clin. Infect. Dis..

[16] National Institute for Biological Standards and Control (NIBSC). https://www.nibsc.org/products/brm_product_catalogue/detail_page.aspx?catid=20/146.

[17] Poljak M., Korva M., Gašper N.K., Komloš K.F., Sagadin M., Uršič T., Županc T.A., Petrovec M. (2020). Clinical evaluation of the cobas SARS-CoV-2 test and a diagnostic platform switch during 48 h in the midst of the COVID-19 pandemic. J. Clin. Microbiol..

[18] You H.L., Lin M.C., Lee C.H. (2021). Comparison of the Roche cobas 6800 SARS-CoV-2 test and the Taiwan CDC protocol for the molecular diagnosis of COVID-19. Biomed. J..

